# Supplementary material for: Molecular Dynamics Simulations of Drug-Conjugated Cell-Penetrating Peptides
Source: Pharmaceuticals (Basel). 2023 Sep 5;16(9):1251. doi: 10.3390/ph16091251 (PMC10535489; doi:10.3390/ph16091251)
Supplement: Supplementary file 1 [file pharmaceuticals-16-01251-s001.zip › pharmaceuticals-2482591-supplementary.pdf]

Supporting Information: Molecular dynamics simulations of drug-conjugated cell-penetrating peptides

Márton Ivánczi, Balazs Balogh, Loretta Kis and István Mándity *

Department of Organic Chemistry, Semmelweis University, H-1089 Üllői út 26, Budapest, Hungary; mandity.istvan@pharma.semmelweis-univ.hu

Table S1. Comprehensive list of the simulations. All of the simulation boxes contained POPC membrane model, the peptide conjugates were placed in the water box above the membrane in parallel position. The simulation time always was 1000 ns and the temperature was 300 K.

**Peptide**	**Conjugate**
1KZ0	unconjugated
1KZ0	doxorubicin
1KZ0	rasagiline
1KZ0	zidovudine
1KZ2	unconjugated
1KZ2	doxorubicin
1KZ2	rasagiline
1KZ2	zidovudine
1KZ5	unconjugated
1KZ5	doxorubicin
1KZ5	rasagiline
1KZ5	zidovudine
1NB1	unconjugated
1NB1	doxorubicin
1NB1	rasagiline
1NB1	zidovudine
1JBL	unconjugated

1JBL	doxorubicin
1JBL	rasagiline
1JBL	zidovudine
1HA9	unconjugated
1HA9	doxorubicin
1HA9	rasagiline
1HA9	zidovudine

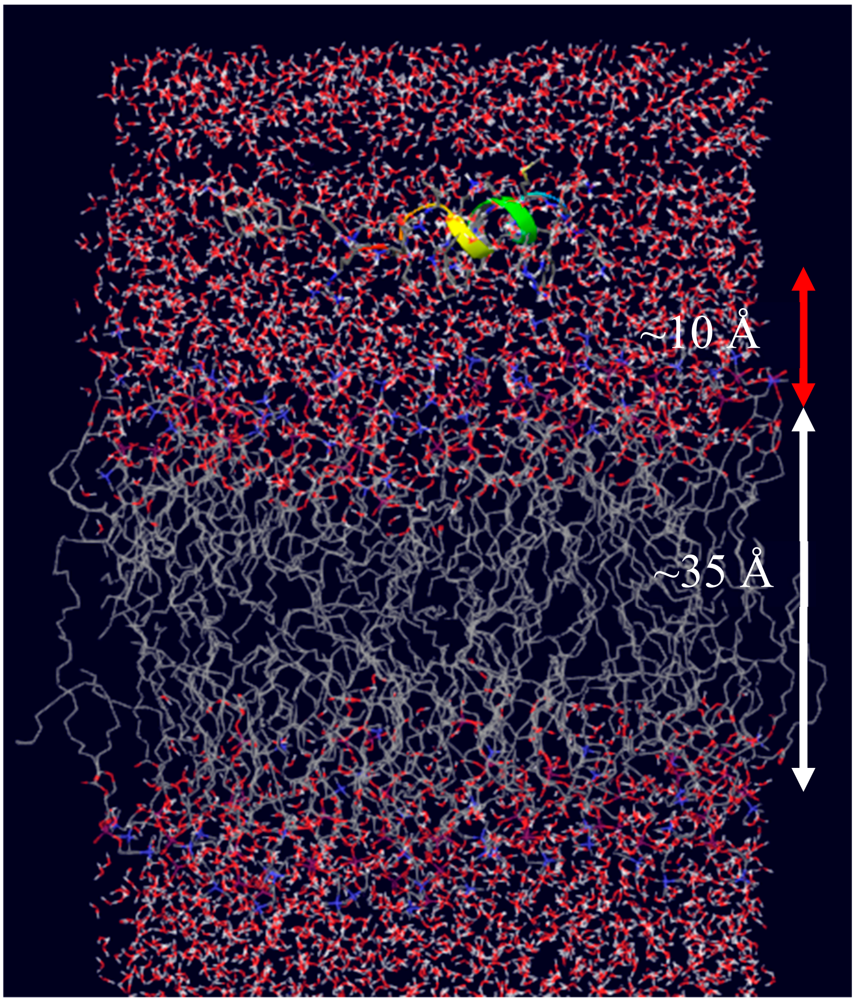

Figure S1. The arrangement of the MD simulation systems: starting from the top of the phosphatidylcholine (POPC) membrane bilayer.

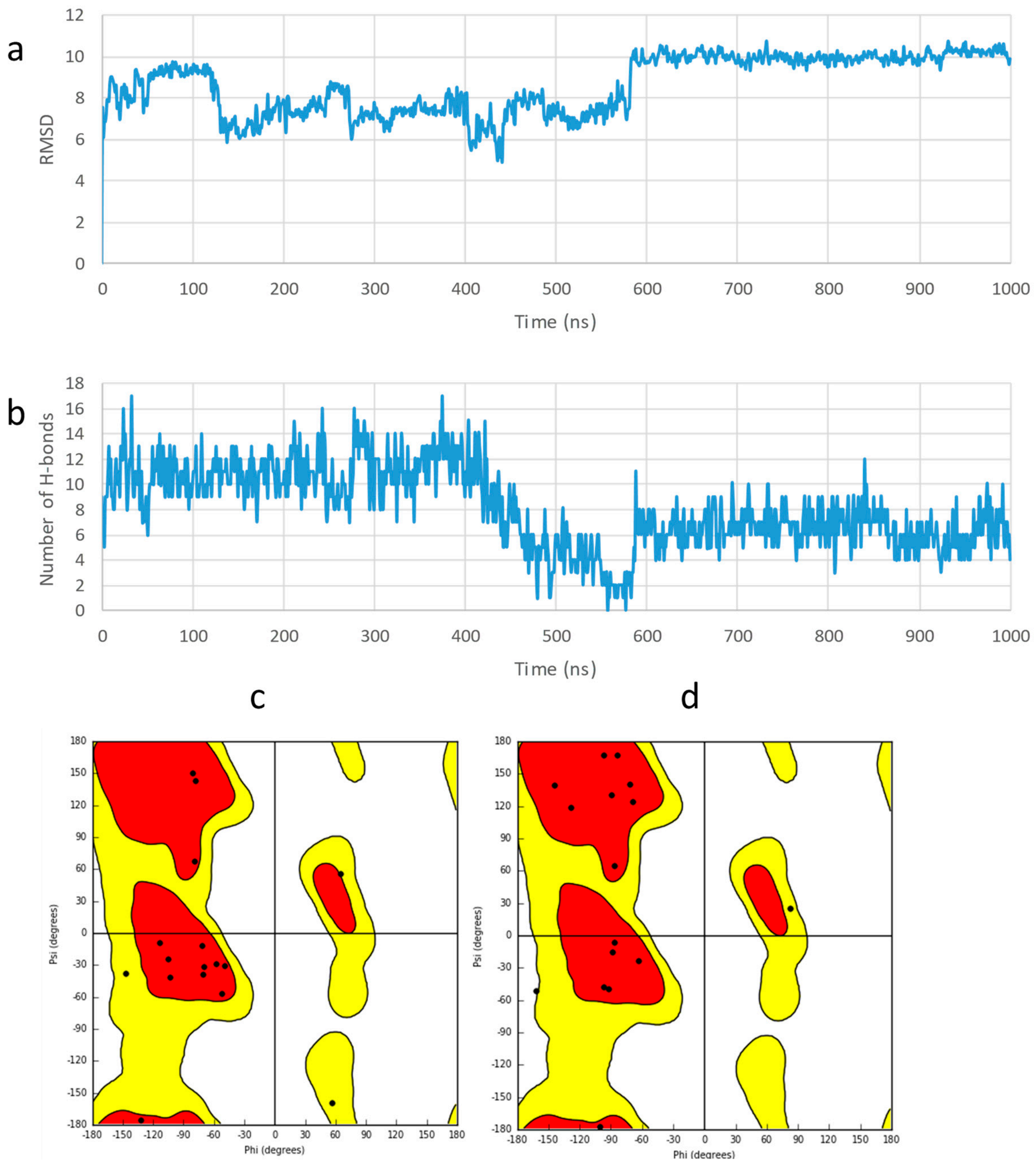

Figure S2. The 1000-ns MD simulation of the unconjugated penetratin (PDB ID: 1KZ0) in the POPC membrane model: the RMSD of α the carbon atoms plotted against the simulation time (a), the number of hydrogen bonds within the peptide plotted against the simulation time (b), the Ramachandran plot of the peptide at 0 ns (c) and at 1000 ns (d) – starting from the top of the membrane.

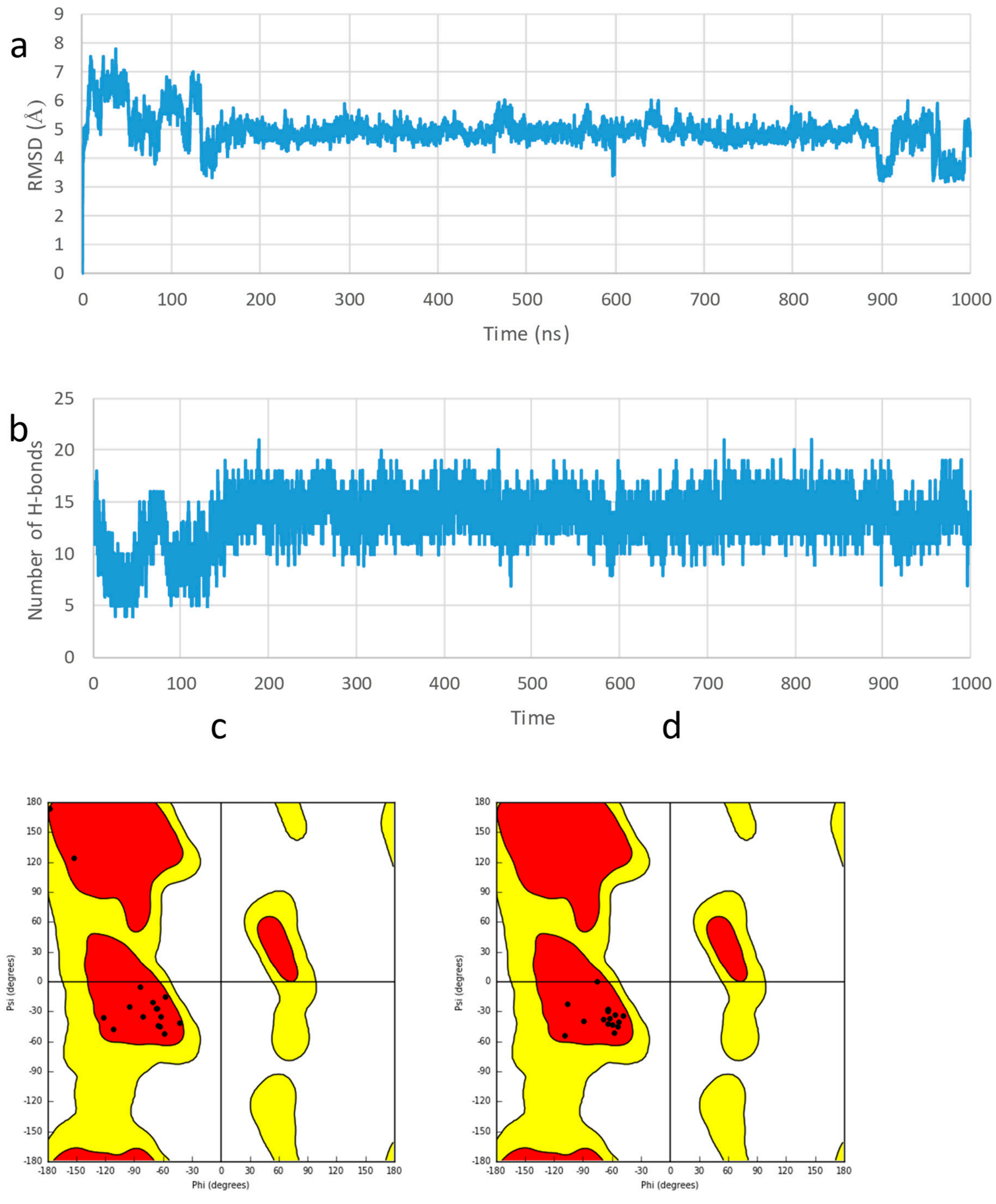

Figure S3. The 1000-ns MD simulation of the unconjugated 6,14-Phe-penetratin (PDB ID: 1KZ2) in the POPC membrane model: the RMSD of the α-carbon atoms plotted against the simulation time (a), the number of hydrogen bonds within the peptide plotted against the simulation time (b), the Ramachandran plot of the peptide at 0 ns (c) and at 1000 ns (d) – starting from the top of the membrane.

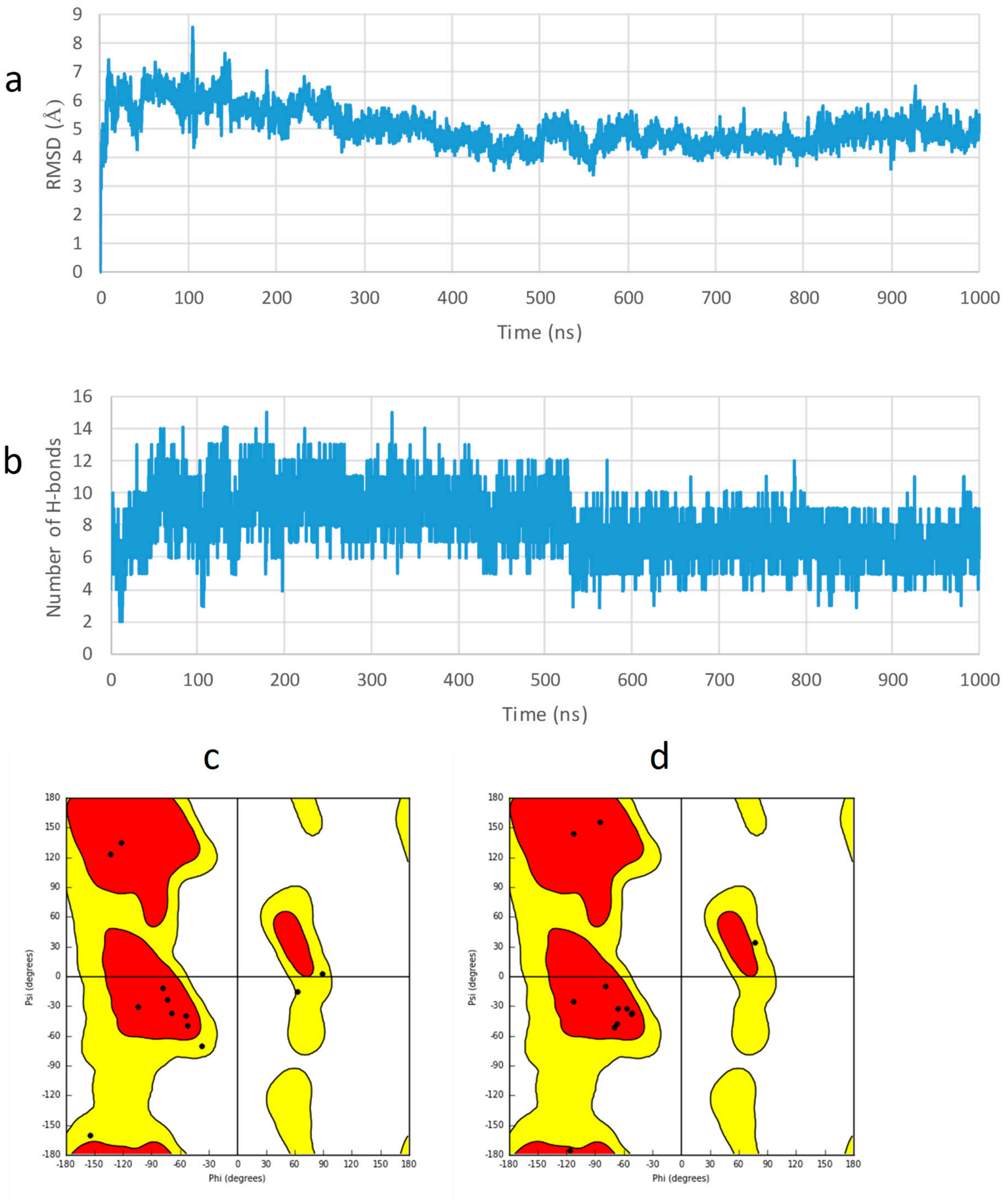

Figure S4. The 1000-ns MD simulation of the unconjugated dodeca-penetratin (PDB ID: 1KZ5) in the POPC membrane model: the RMSD of the α-carbon atoms plotted against the simulation time (a), the number of hydrogen bonds within the peptide plotted against the simulation time (b), the Ramachandran plot of the peptide at 0 ns (c) and at 1000 ns (d) – starting from the top of the membrane.

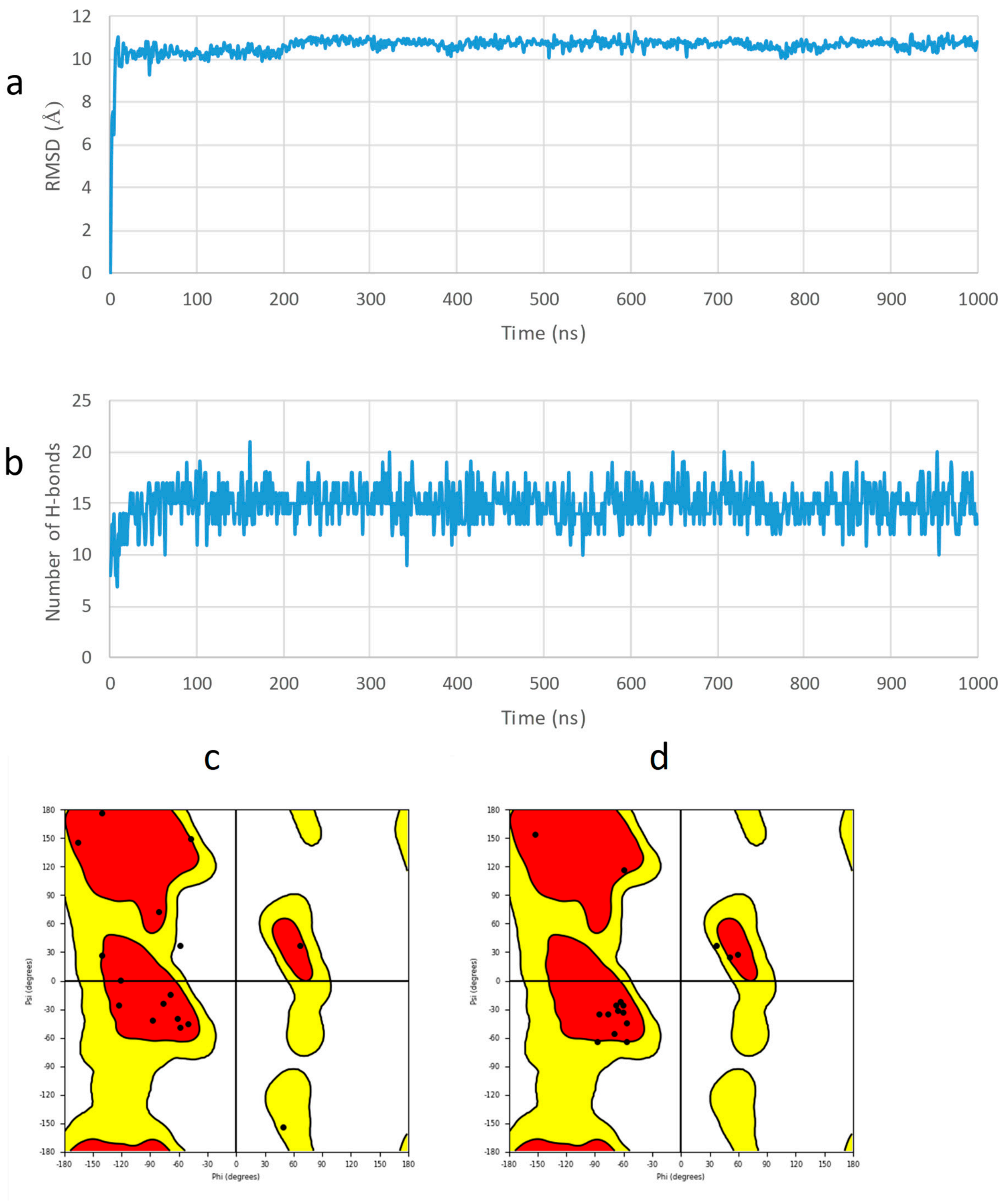

Figure S5. The 1000-ns MD simulation of the penetratin–doxorubicin conjugate in the POPC membrane model: the RMSD of the α-carbon atoms plotted against the simulation time (a), the number of hydrogen bonds within the peptide plotted against the simulation time (b), the Ramachandran plot of the peptide at 0 ns (c) and at 1000 ns (d) – starting from the top of the membrane.

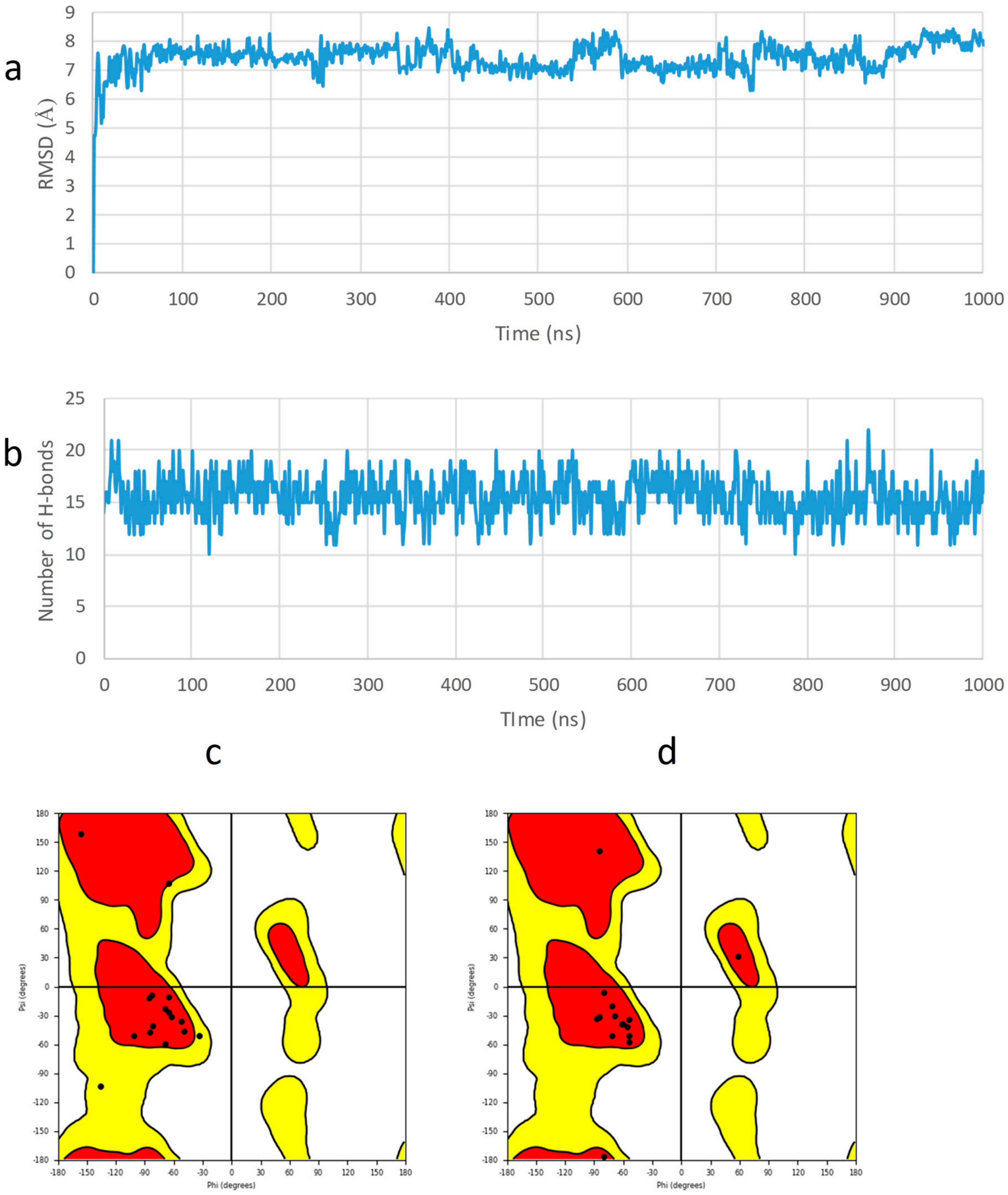

Figure S6. The 1000-ns MD simulation of the of 6,14-Phe-penetratin–doxorubicin conjugate in the POPC membrane model: the RMSD of the α-carbon atoms plotted against the simulation time (a), the number of hydrogen bonds within the peptide plotted against the simulation time (b), the Ramachandran plot of the peptide at 0 ns (c) and at 1000 ns (d) – starting from the top of the membrane.

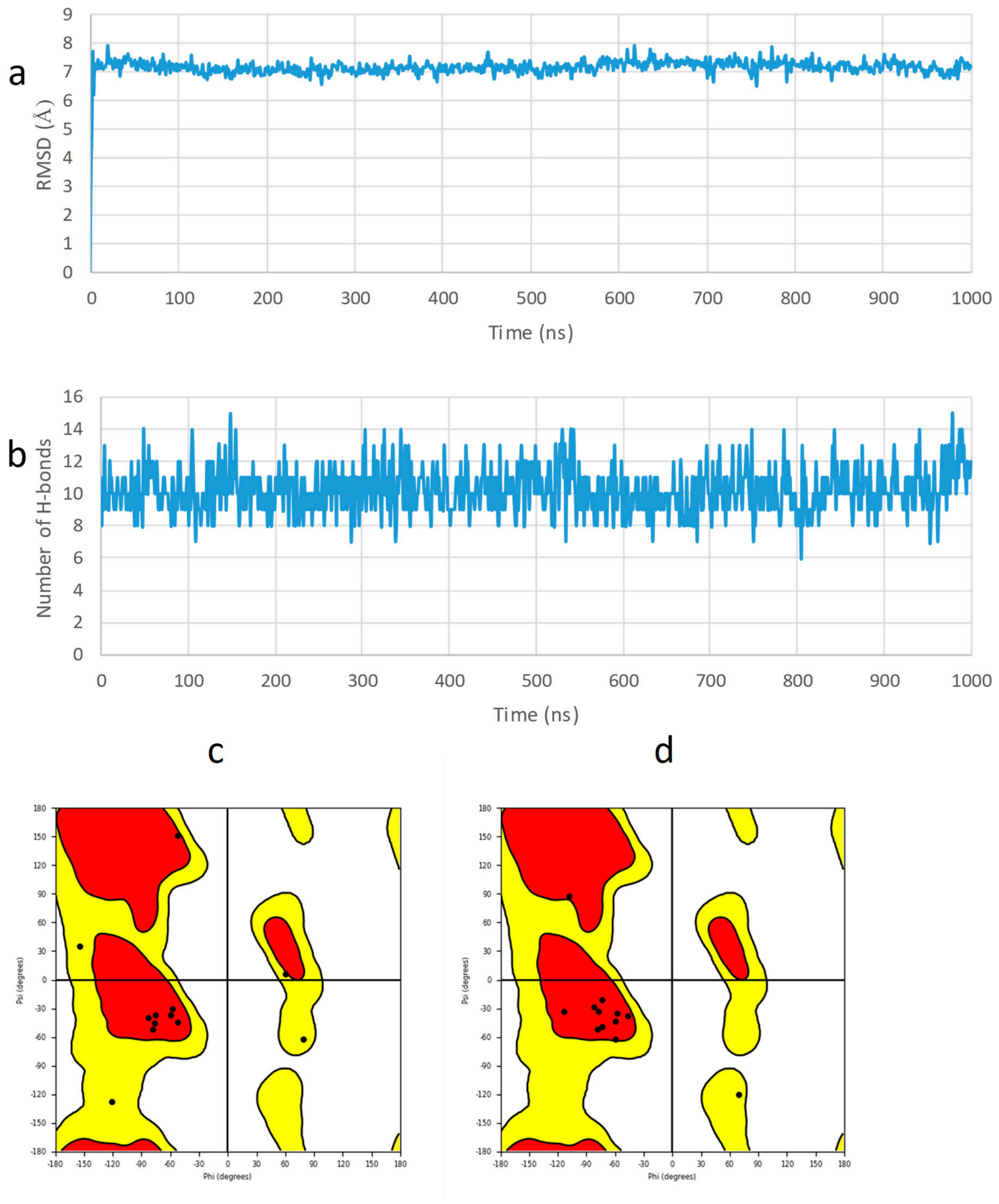

Figure S7. The 1000-ns MD simulation of the of dodeca-penetratin–doxorubicin conjugate in the POPC membrane model: the RMSD of the α-carbon atoms plotted against the simulation time (a), the number of hydrogen bonds within the peptide plotted against the simulation time (b), the Ramachandran plot of the peptide at 0 ns (c) and at 1000 ns (d) – starting from the top of the membrane.

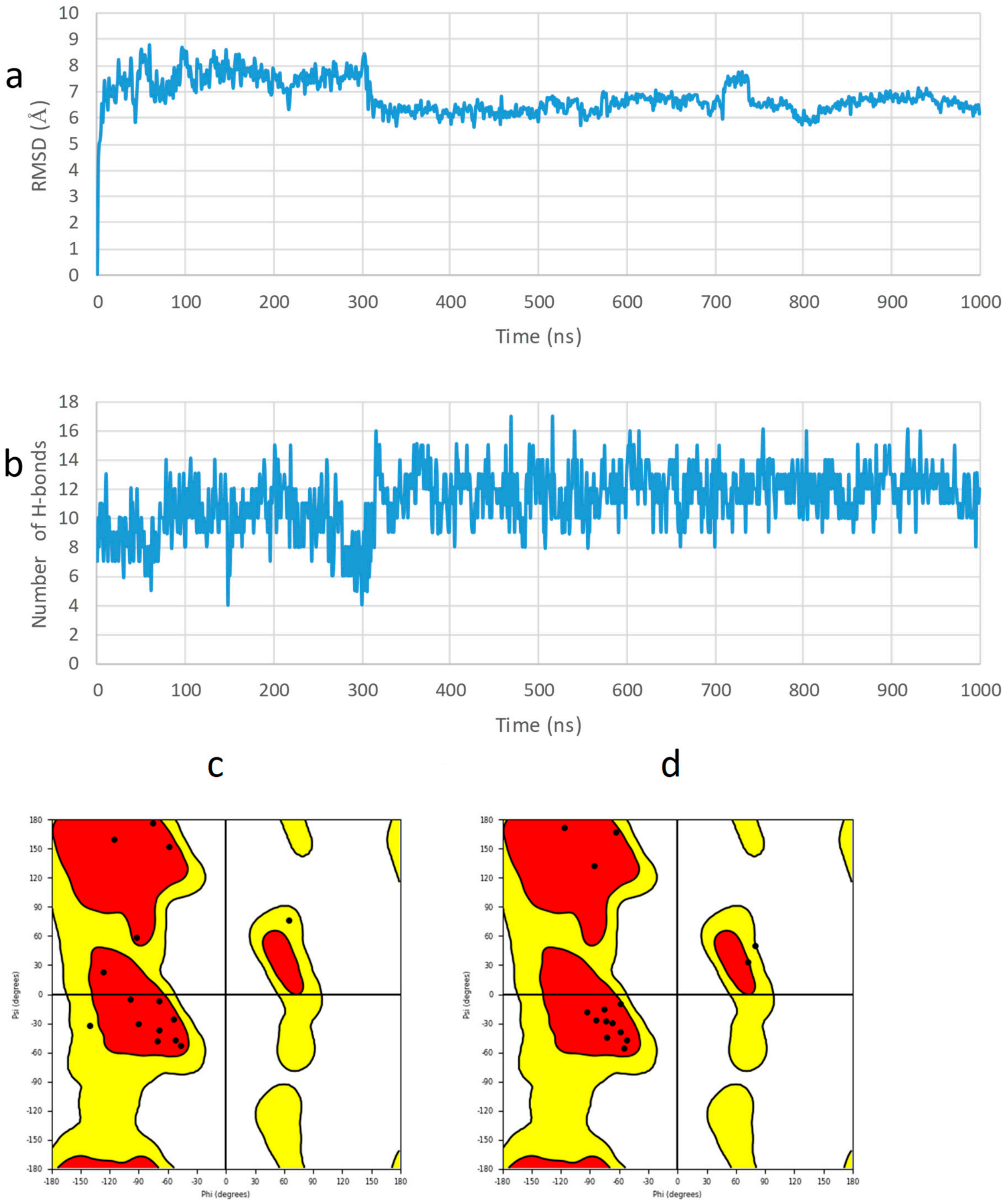

Figure S8. The 1000 ns MD simulation of the penetratin–rasagiline conjugate in the POPC membrane model: the RMSD of the α-carbon atoms plotted against the simulation time (a), the number of hydrogen bonds within the peptide plotted against the simulation time (b), the Ramachandran plot of the peptide at 0 ns (c) and at 1000 ns (d) – starting from the top of the membrane.

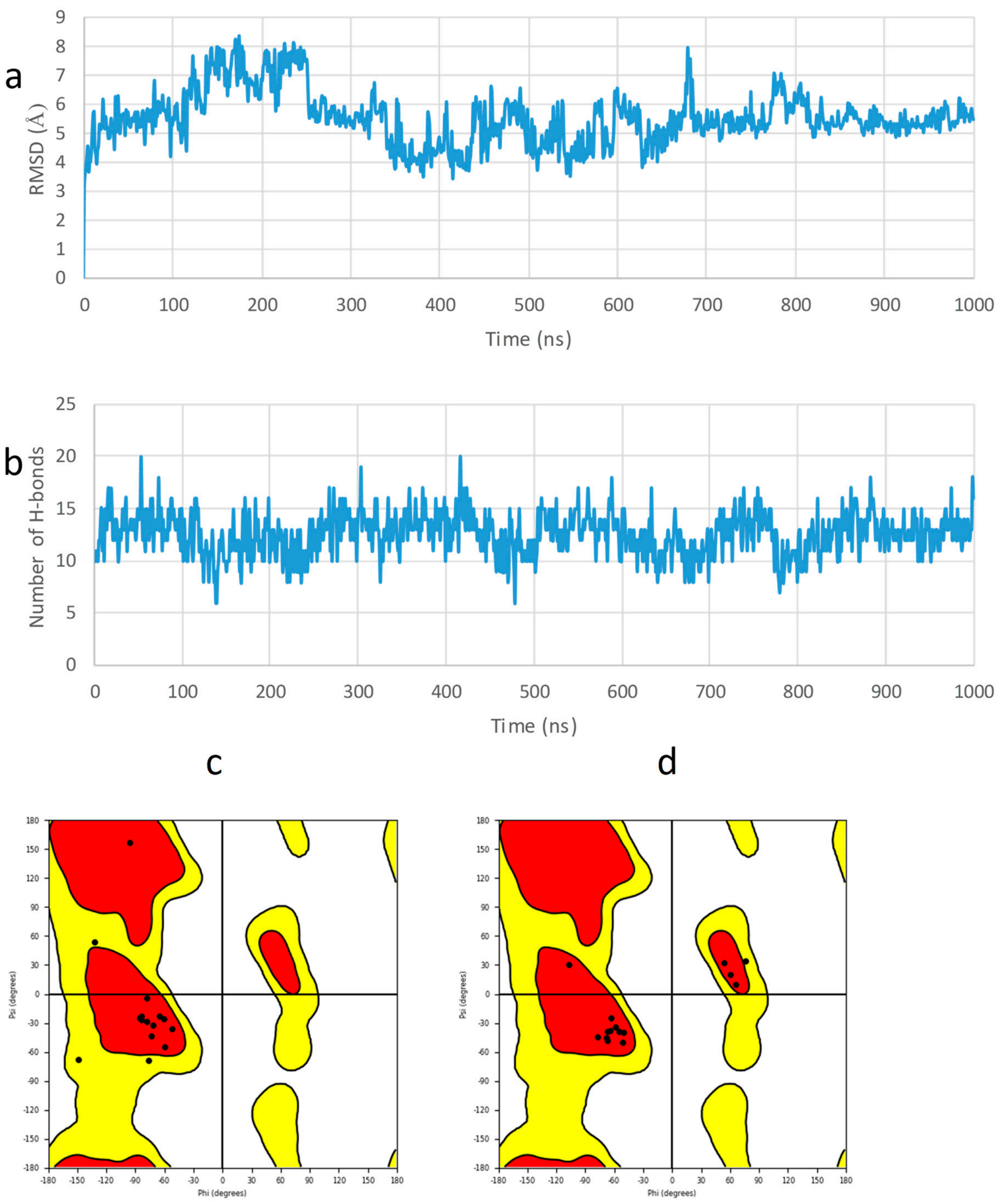

Figure S9. The 1000-ns MD simulation of the of 6,14-Phe-penetratin–rasagiline conjugate in the POPC membrane model: the RMSD of the α-carbon atoms plotted against the simulation time (a), the number of hydrogen bonds within the peptide plotted against the simulation time (b), the Ramachandran plot of the peptide at 0 ns (c) and at 1000 ns (d) – starting from the top of the membrane.

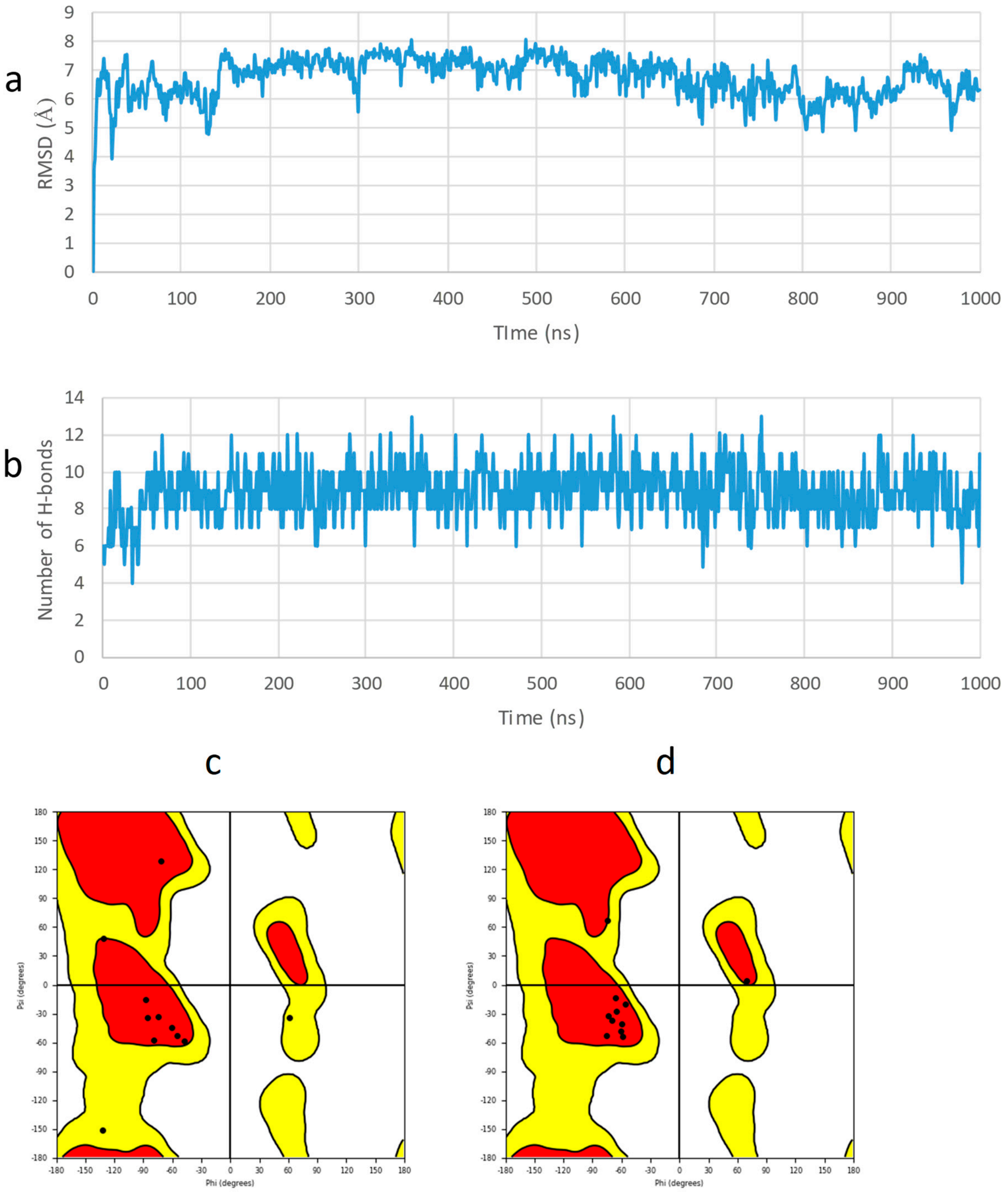

Figure S10. The 1000-ns MD simulation of the of the dodeca-penetratin–rasagiline conjugate in the POPC membrane model: the RMSD of α the carbon atoms plotted against the simulation time (a), the number of hydrogen bonds within the peptide plotted against the simulation time (b), the Ramachandran plot of the peptide at 0 ns (c) and at 1000 ns (d) – starting from the top of the membrane.

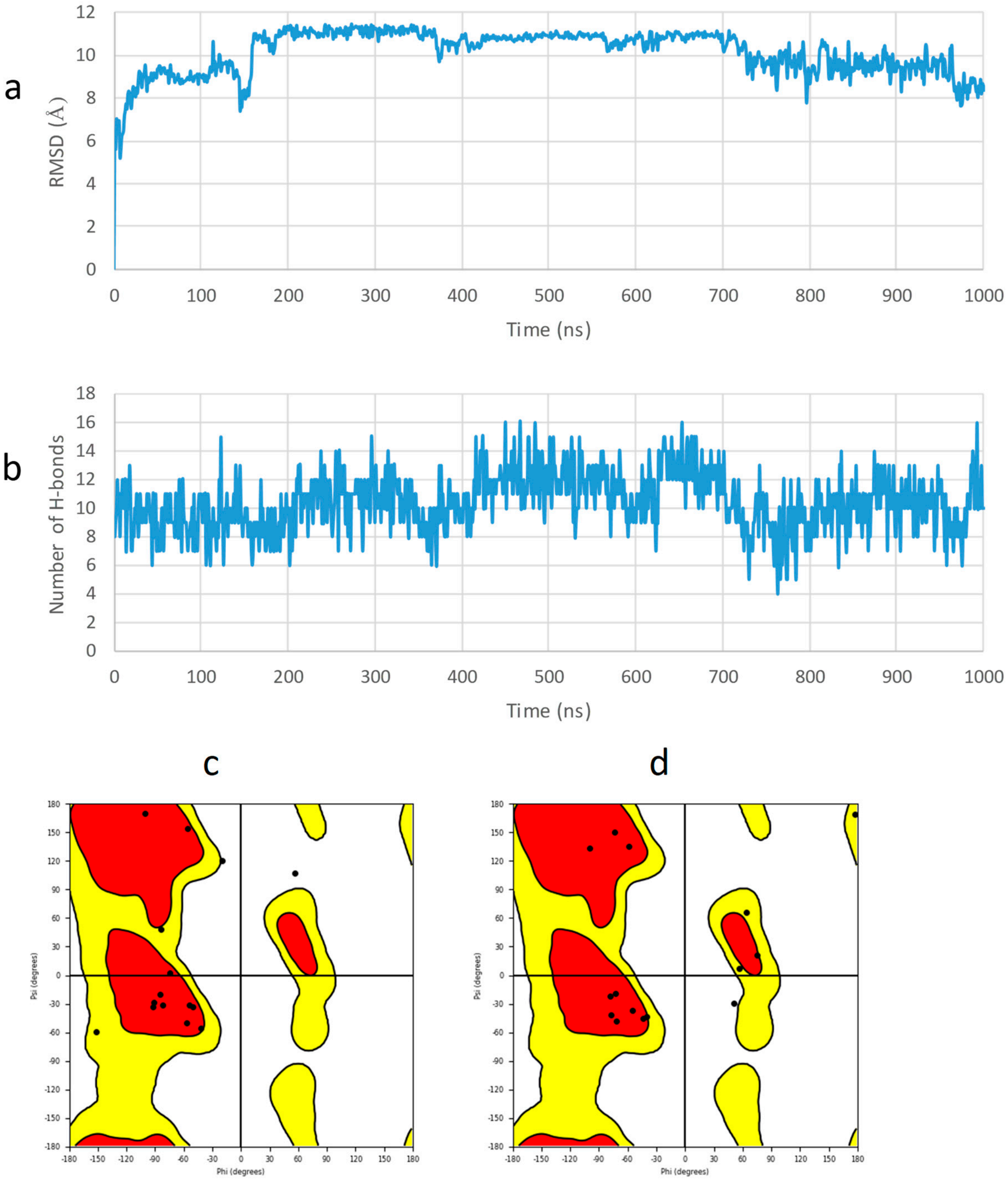

Figure S11. The 1000-ns MD simulation of the penetratin–zidovudine conjugate in the POPC membrane model: the RMSD of the α-carbon atoms plotted against the simulation time (a), the number of hydrogen bonds within the peptide plotted against the simulation time (b), the Ramachandran plot of the peptide at 0 ns (c) and at 1000 ns (d) – starting from the top of the membrane.

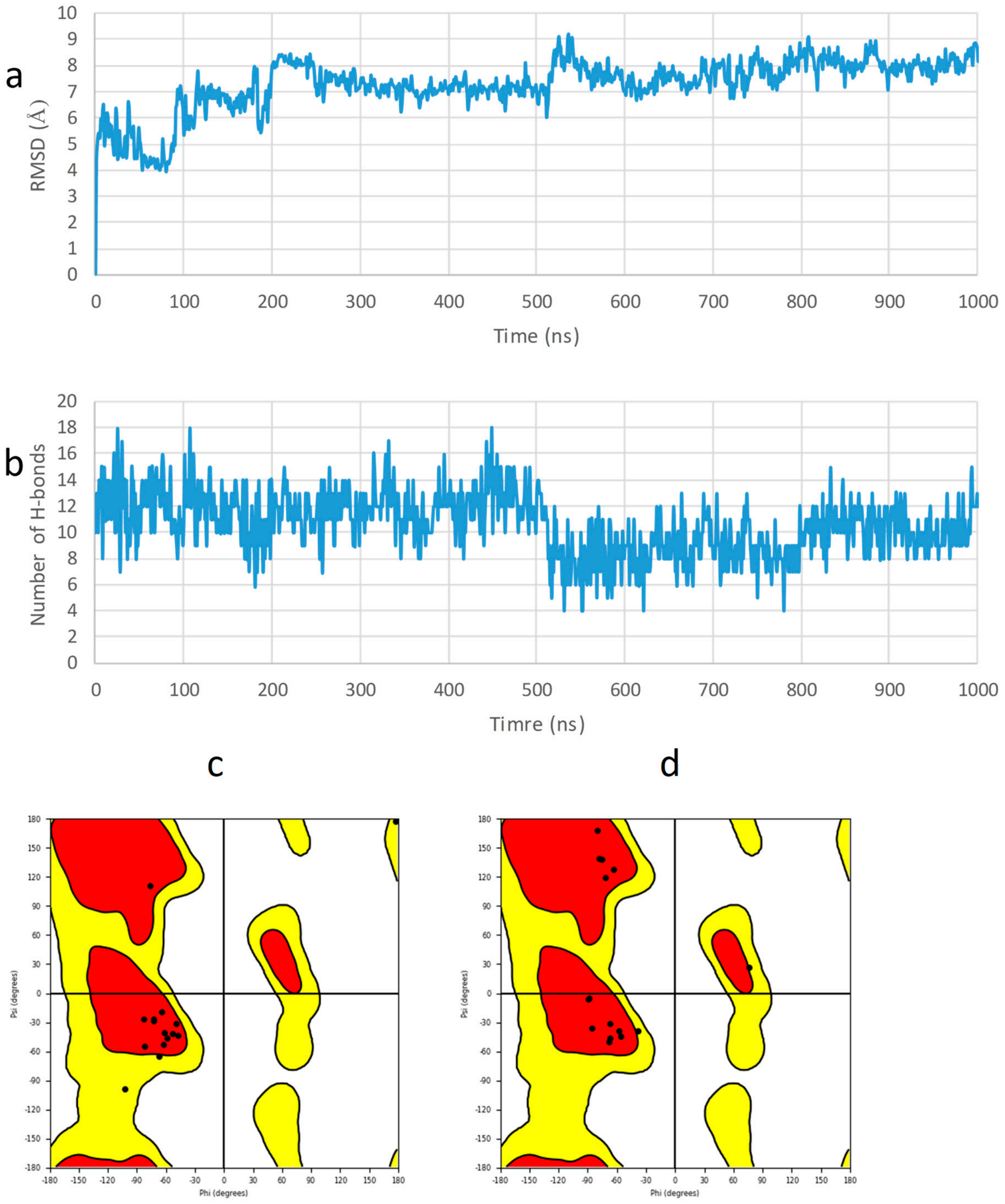

Figure S12. The 1000-ns MD simulation of the 6,14-Phe-penetratin–zidovudine conjugate in the POPC membrane model: the RMSD of α the carbon atoms plotted against the simulation time (a), the number of hydrogen bonds within the peptide plotted against the simulation time (b), the Ramachandran plot of the peptide at 0 ns (c) and at 1000 ns (d) – starting from the top of the membrane.

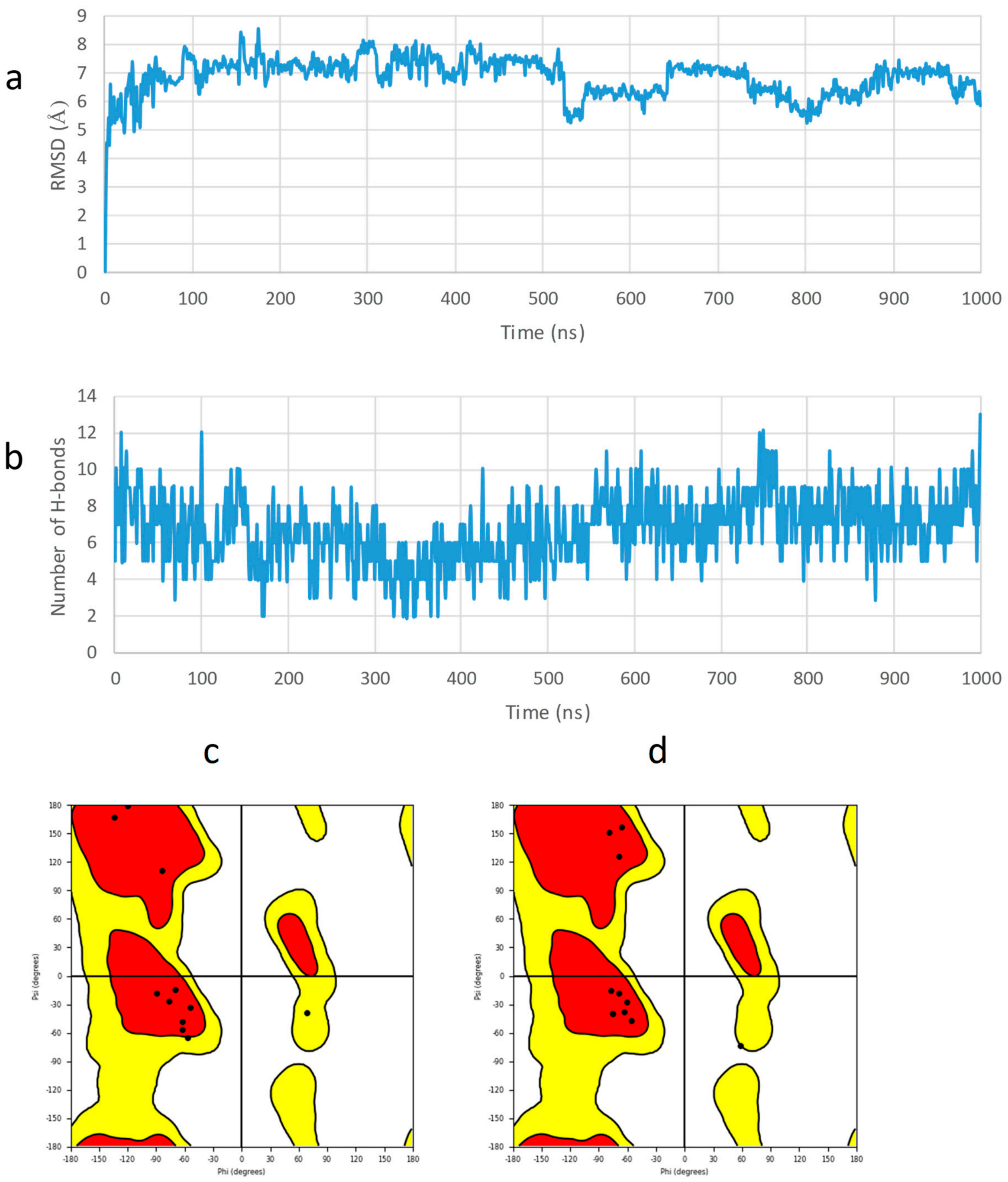

Figure S13. The 1000-ns MD simulation of the dodeca-penetratin–zidovudine conjugate in the POPC membrane model: the RMSD of α the carbon atoms plotted against the simulation time (a), the number of hydrogen bonds within the peptide plotted against the simulation time (b), the Ramachandran plot of the peptide at 0 ns (c) and at 1000 ns (d) – starting from the top of the membrane.

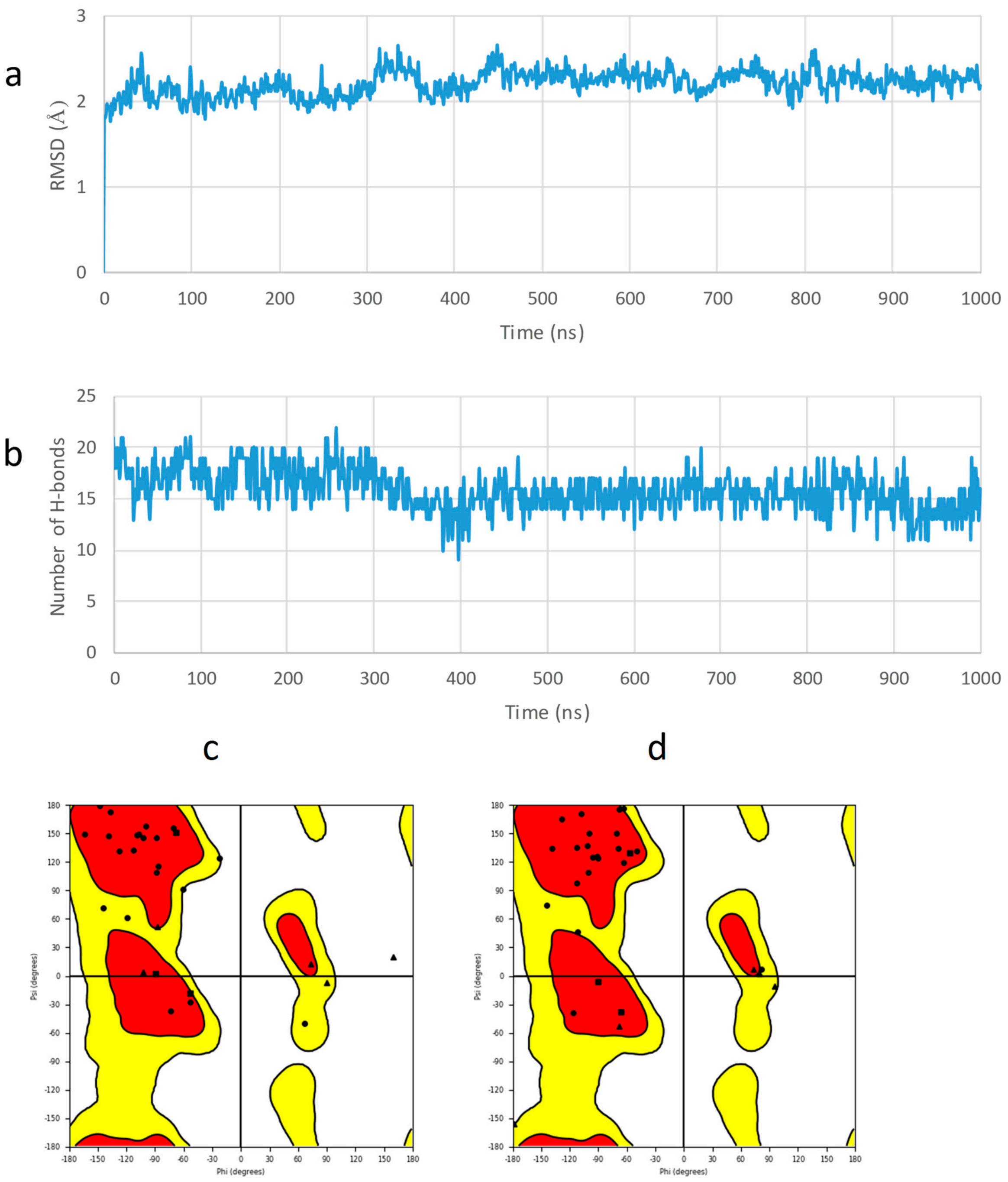

Figure S14. The 1000-ns MD simulation of the unconjugated Kalata B1 (PDB ID: 1NB1) in the POPC membrane model: the RMSD of the α-carbon atoms plotted against the simulation time (a), the number of hydrogen bonds within the peptide plotted against the simulation time (b), the Ramachandran plot of the peptide at 0 ns (c) and at 1000 ns (d) – starting from the top of the membrane.

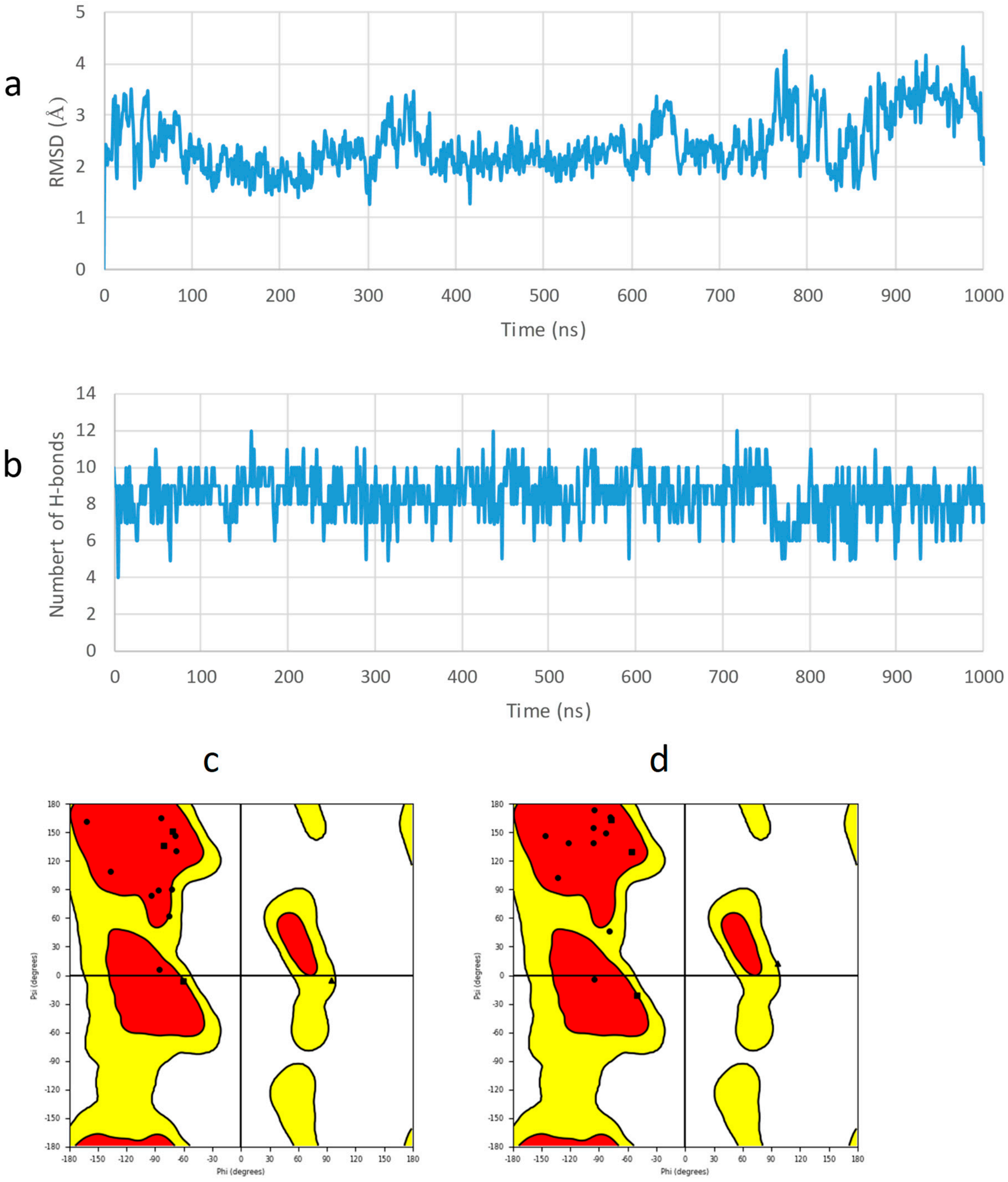

Figure S15. The 1000 ns MD simulation of the unconjugated SFTI-1 (PDB ID: 1JBL) in the POPC membrane model: the RMSD of the α-carbon atoms plotted against the simulation time (a), the number of hydrogen bonds within the peptide plotted against the simulation time (b), the Ramachandran plot of the peptide at 0 ns (c) and at 1000 ns (d) – starting from the top of the membrane.

a

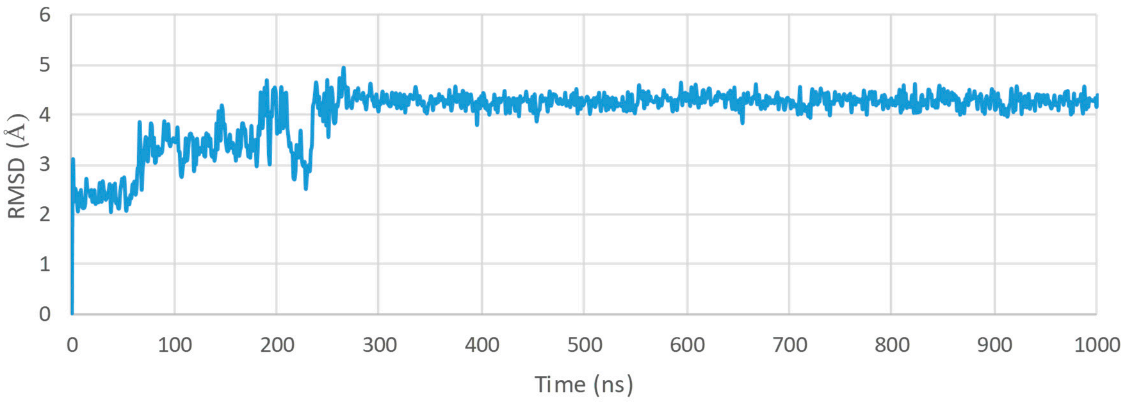

b

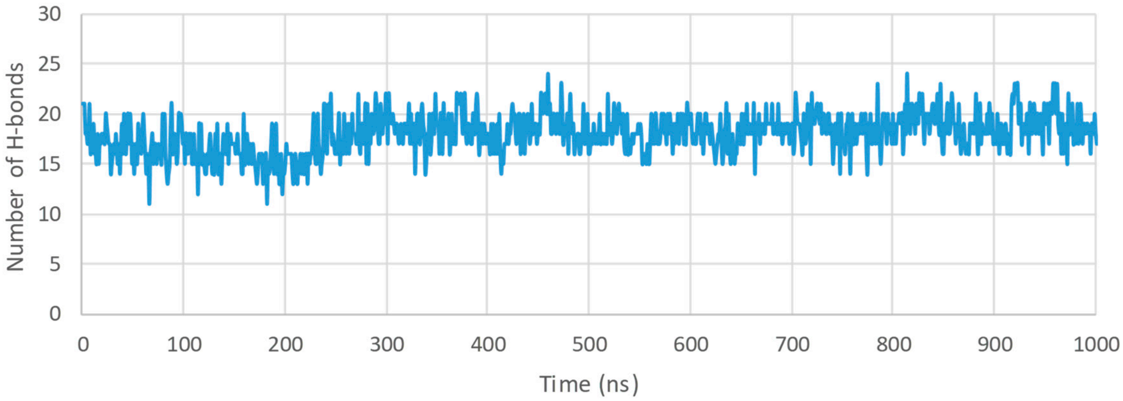

c

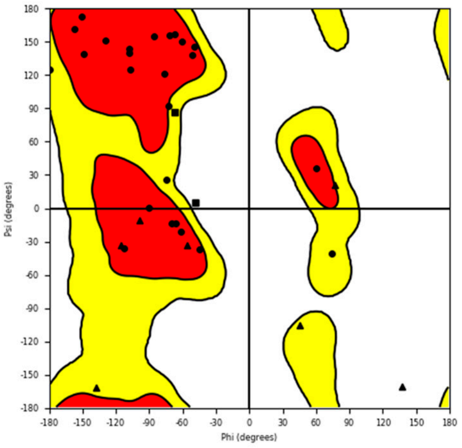

d

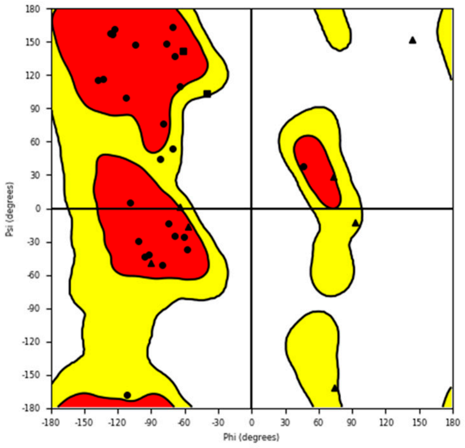

Figure S16. The 1000-ns MD simulation of the unconjugated MCoTI-II (PDB ID: 1HA9) in the POPC membrane model: the RMSD of the α-carbon atoms plotted against the simulation time (a), the number of hydrogen bonds within the peptide plotted against the simulation time (b), the Ramachandran plot of the peptide at 0 ns (c) and at 1000 ns (d) – starting from the top of the membrane.

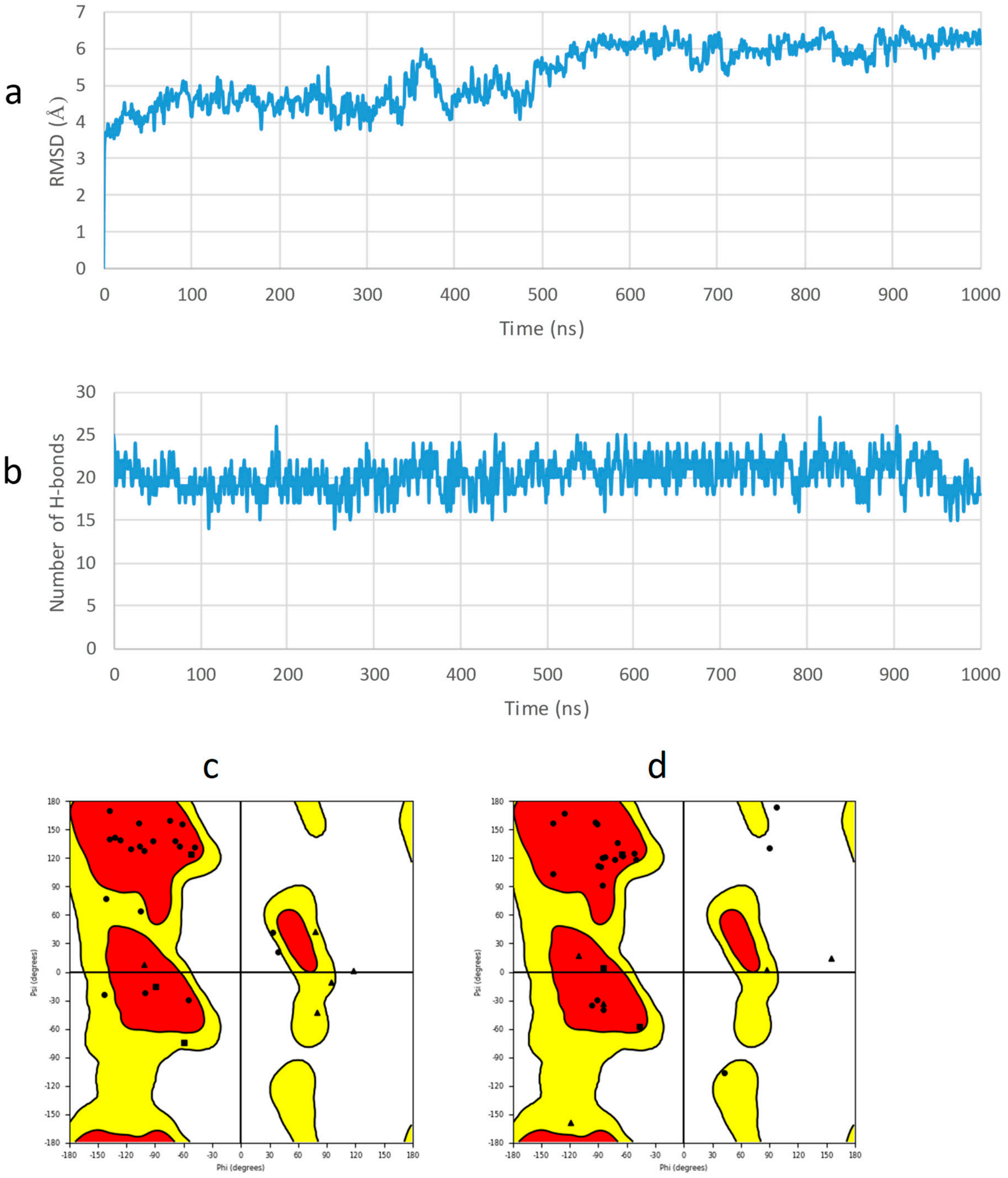

Figure S17. The 1000-ns MD simulation of the Kalata B1–doxorubicin conjugate in P the OPC membrane model: the RMSD of the α-carbon atoms plotted against the simulation time (a), the number of hydrogen bonds within the peptide plotted against the simulation time (b), the Ramachandran plot of the peptide at 0 ns (c) and at 1000 ns (d) – starting from the top of the membrane.

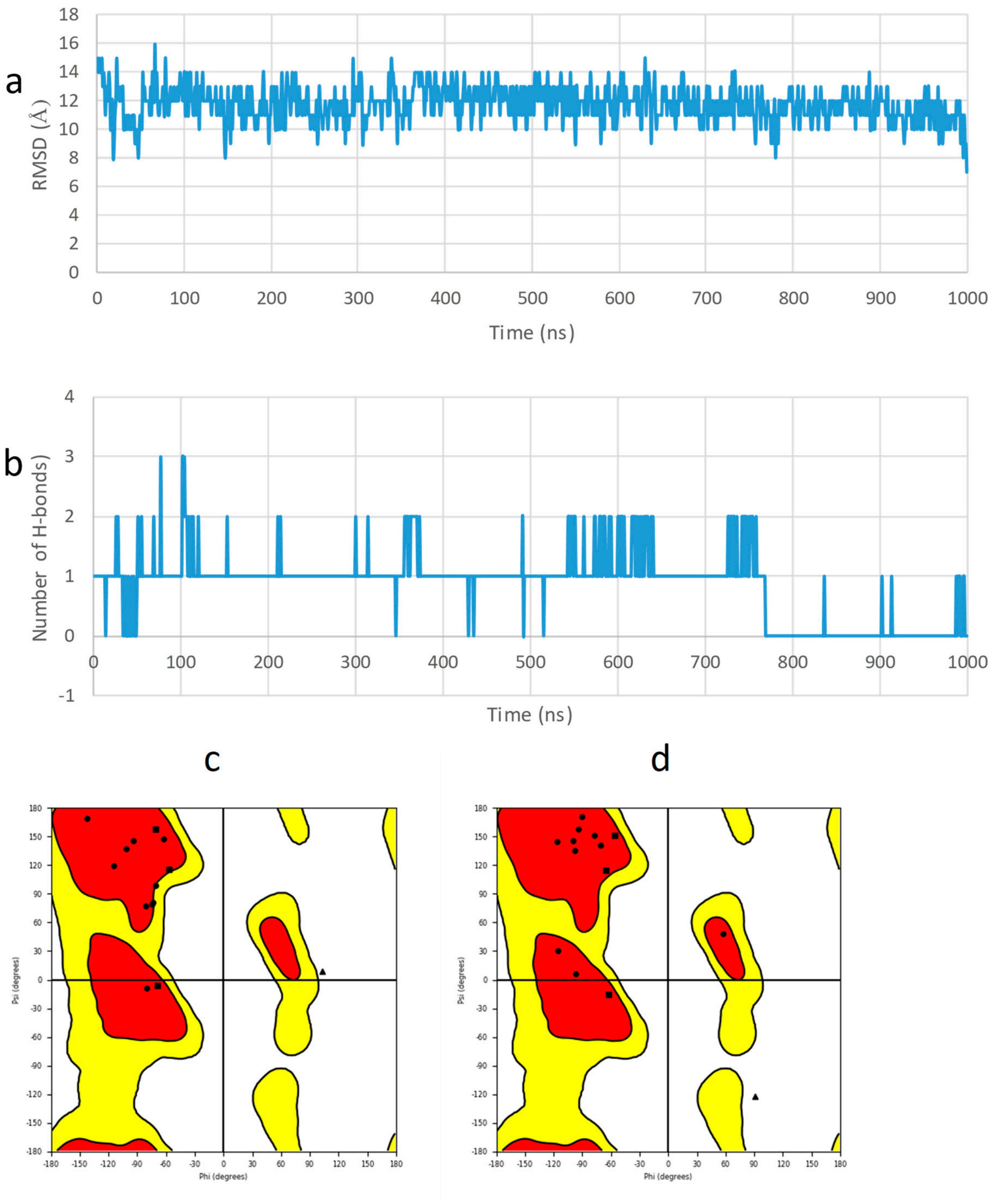

Figure S18. The 1000-ns MD simulation of the SFTI-1–doxorubicin conjugate in the POPC membrane model: the RMSD of the α-carbon atoms plotted against the simulation time (a), the number of hydrogen bonds within the peptide plotted against the simulation time (b), the Ramachandran plot of the peptide at 0 ns (c) and at 1000 ns (d) – starting from the top of the membrane.

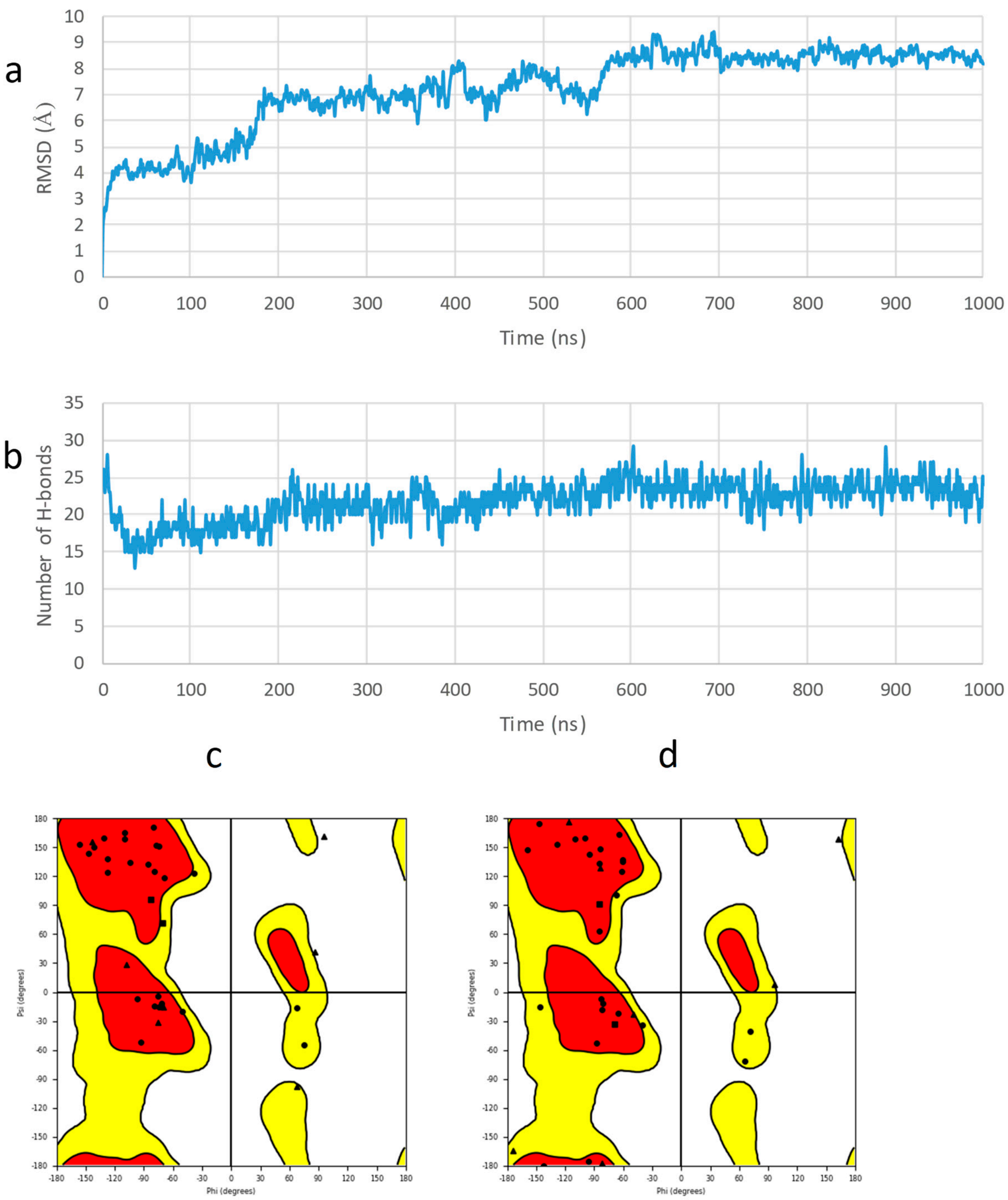

Figure S19. The 1000-ns MD simulation of the MCo-TI-II–doxorubicin conjugate in the POPC membrane model: the RMSD the of α-carbon atoms plotted against the simulation time (a), the number of hydrogen bonds within the peptide plotted against the simulation time (b), the Ramachandran plot of the peptide at 0 ns (c) and at 1000 ns (d) – starting from the top of the membrane.

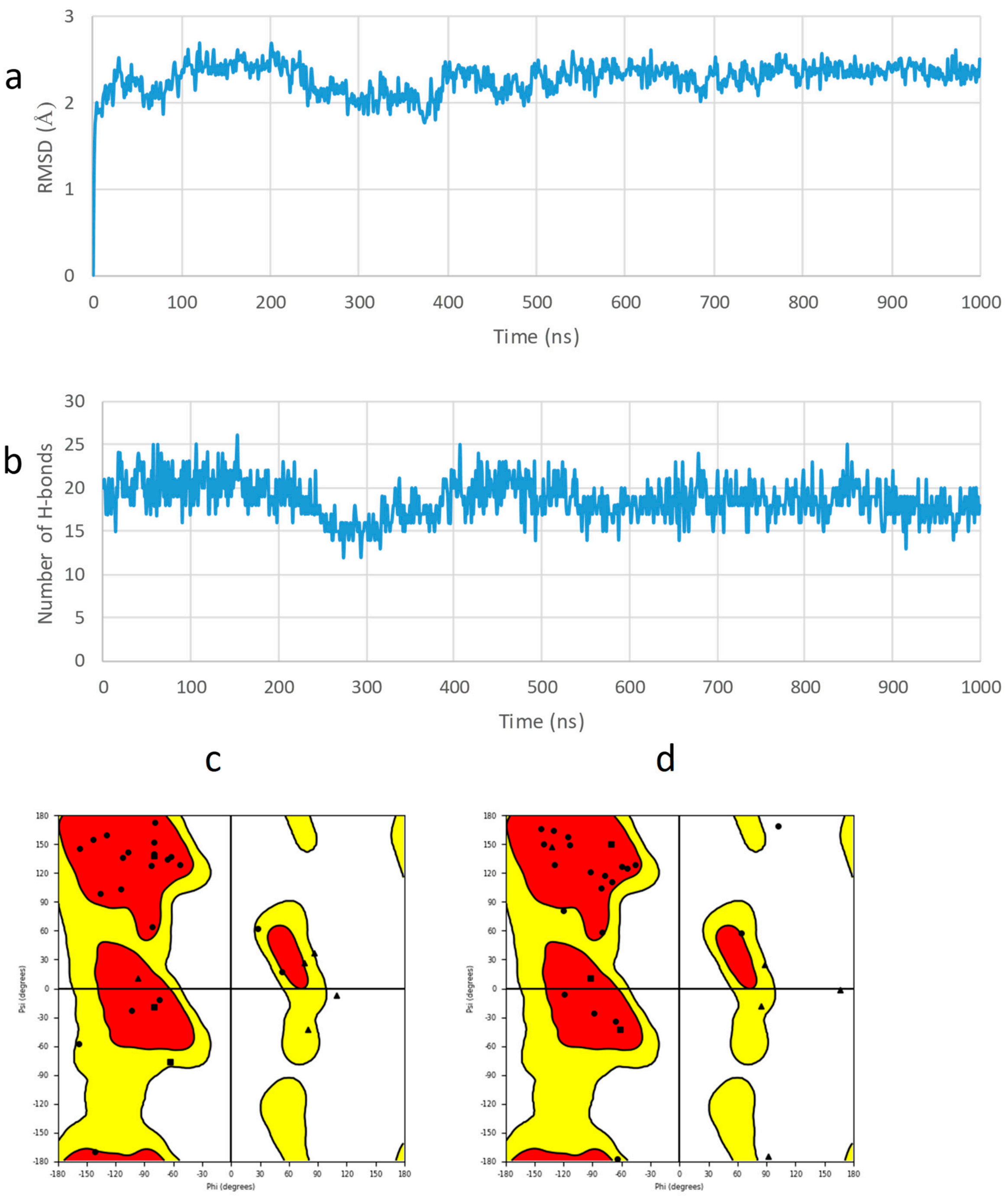

Figure S20. The 1000-ns MD simulation of the Kalata B1–rasagiline conjugate in the POPC membrane model: the RMSD of the α-carbon atoms plotted against the simulation time (a), the number of hydrogen bonds within the peptide plotted against the simulation time (b), the Ramachandran plot of the peptide at 0 ns (c) and at 1000 ns (d) – starting from the top of the membrane.

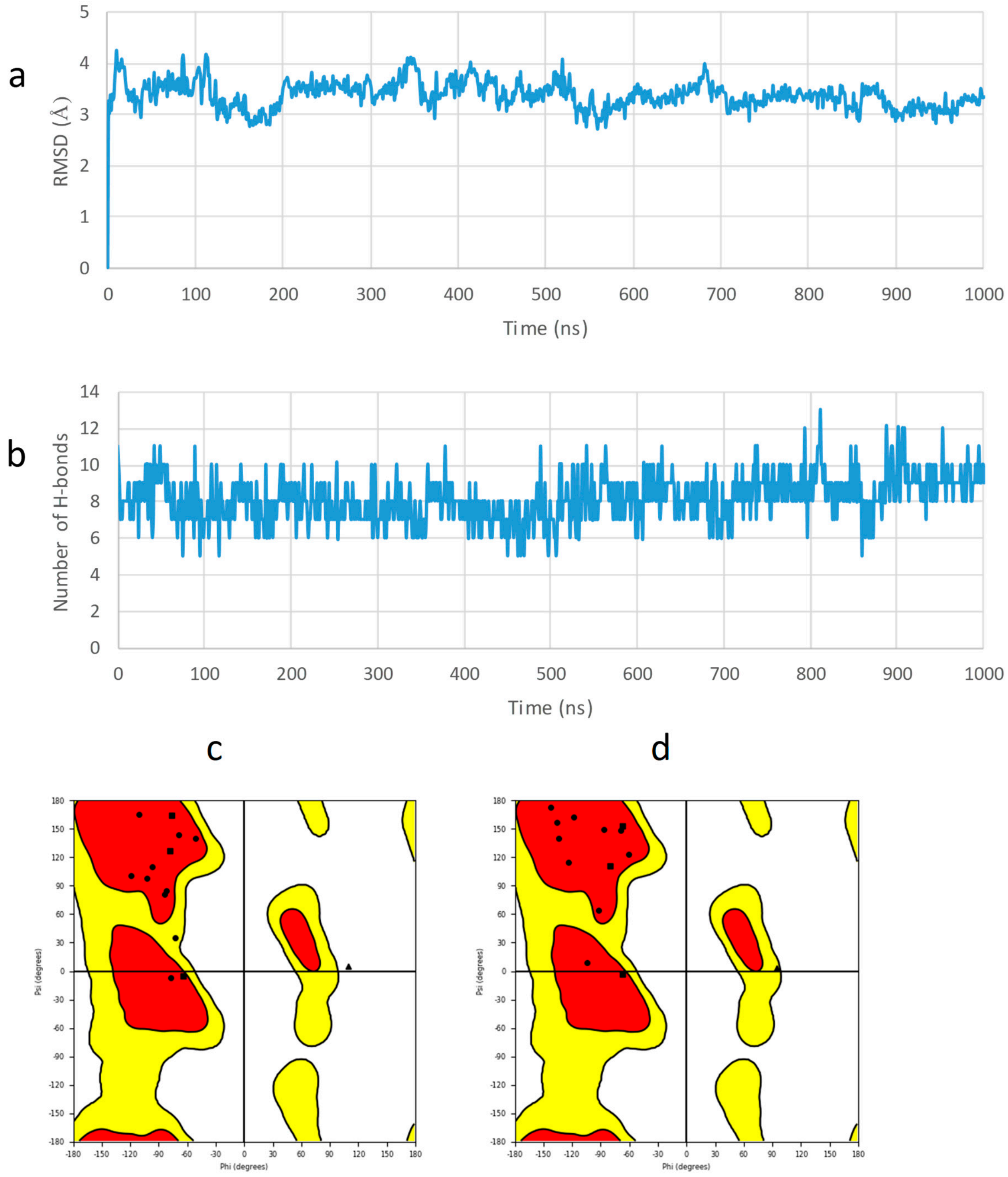

Figure S21. The 1000-ns MD simulation of the SFTI-1–rasagiline conjugate in the POPC membrane model: the RMSD of the α-carbon atoms plotted against the simulation time (a), the number of hydrogen bonds within the peptide plotted against the simulation time (b), the Ramachandran plot of the peptide at 0 ns (c) and at 1000 ns (d) – starting from the top of the membrane.

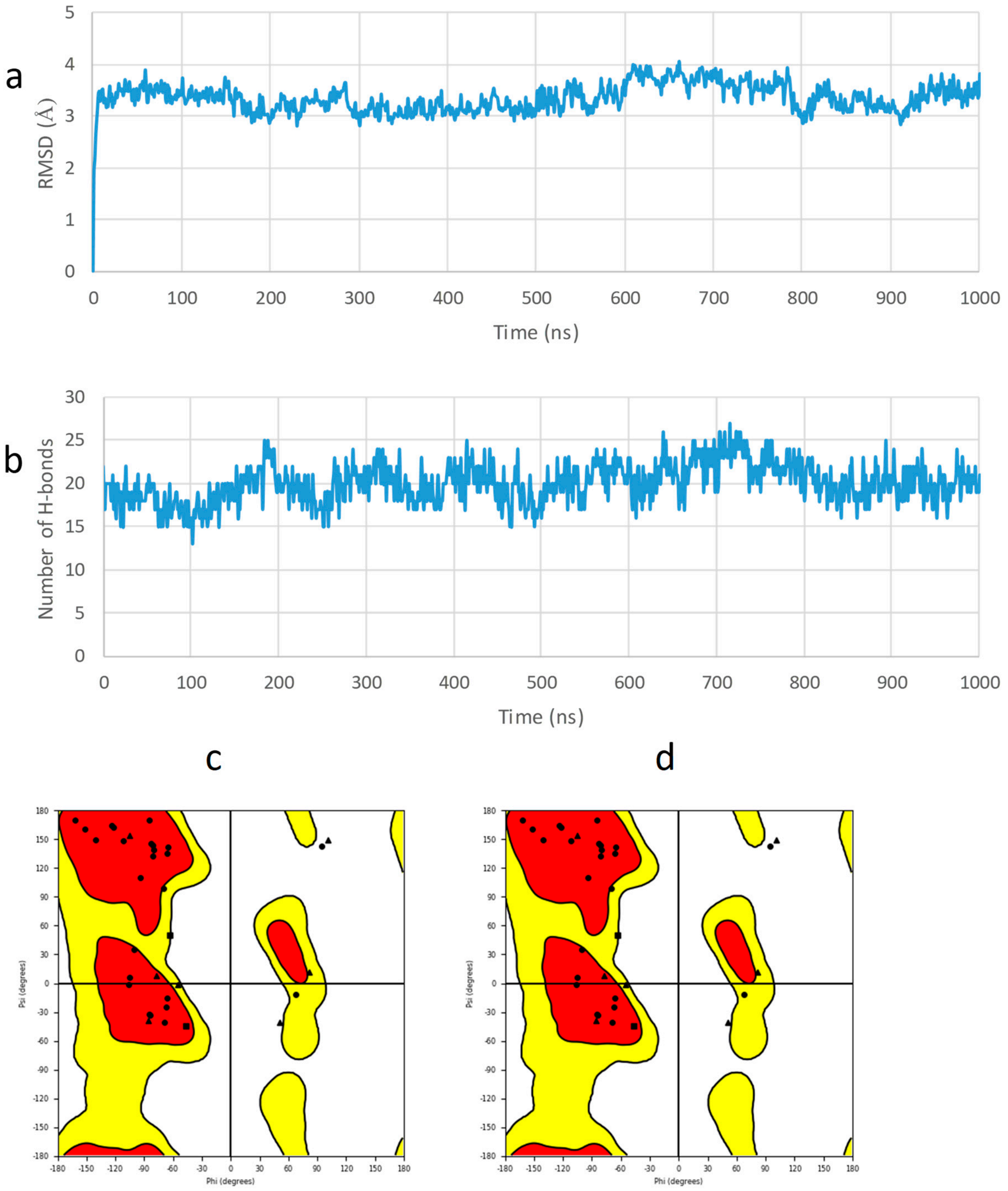

Figure S22. The 1000-ns MD simulation of the MCo-TI-II–rasagiline conjugate in the POPC membrane model: the RMSD of α the carbon atoms plotted against the simulation time (a), the number of hydrogen bonds within the peptide plotted against the simulation time (b), the Ramachandran plot of the peptide at 0 ns (c) and at 1000 ns (d) – starting from the top of the membrane.

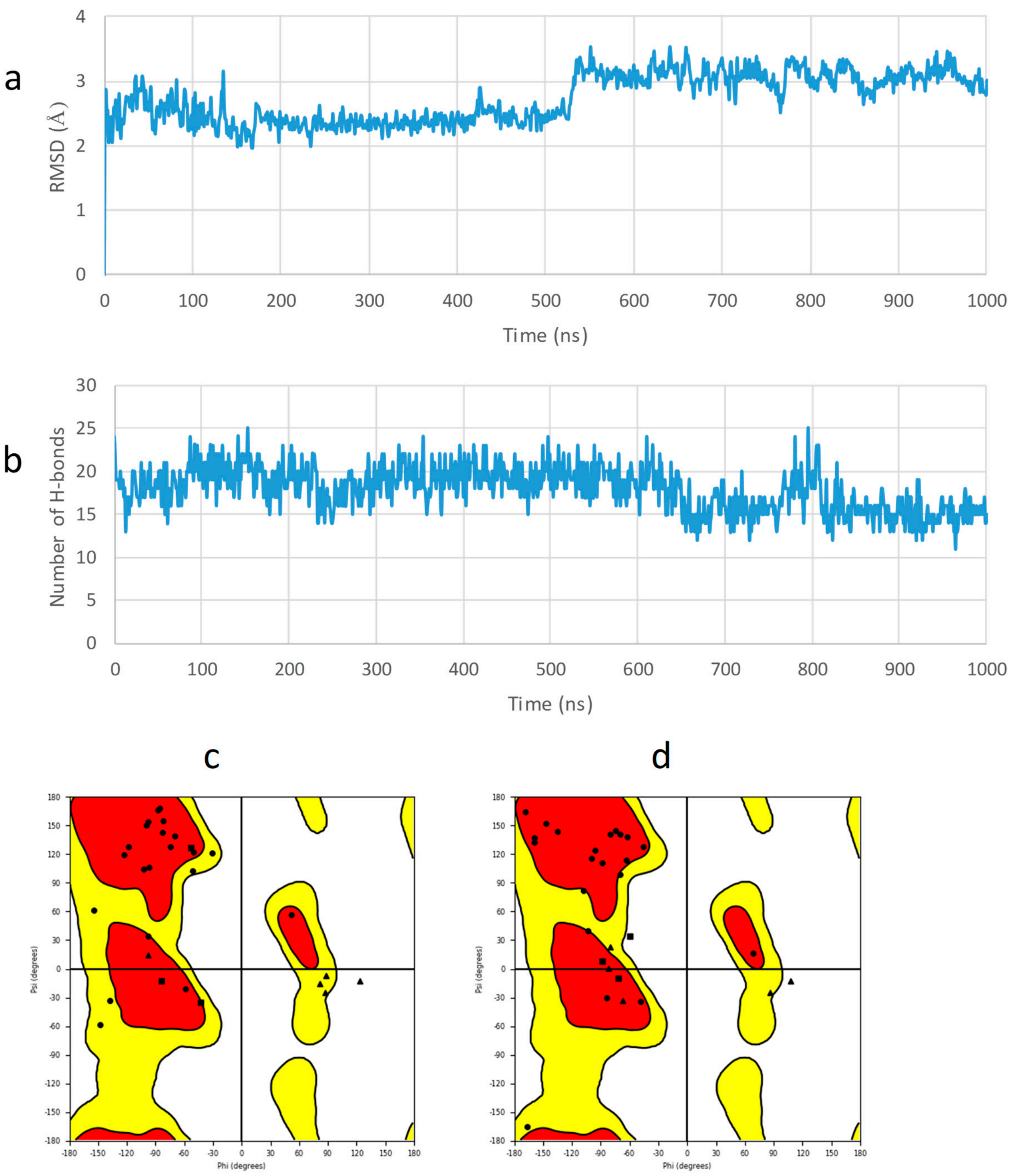

Figure S23. The 1000-ns MD simulation of the Kalata B1–zidovudine conjugate in the POPC membrane model: the RMSD of the α-carbon atoms plotted against the simulation time (a), the number of hydrogen bonds within the peptide plotted against the simulation time (b), the Ramachandran plot of the peptide at 0 ns (c) and at 1000 ns (d) – starting from the top of the membrane.

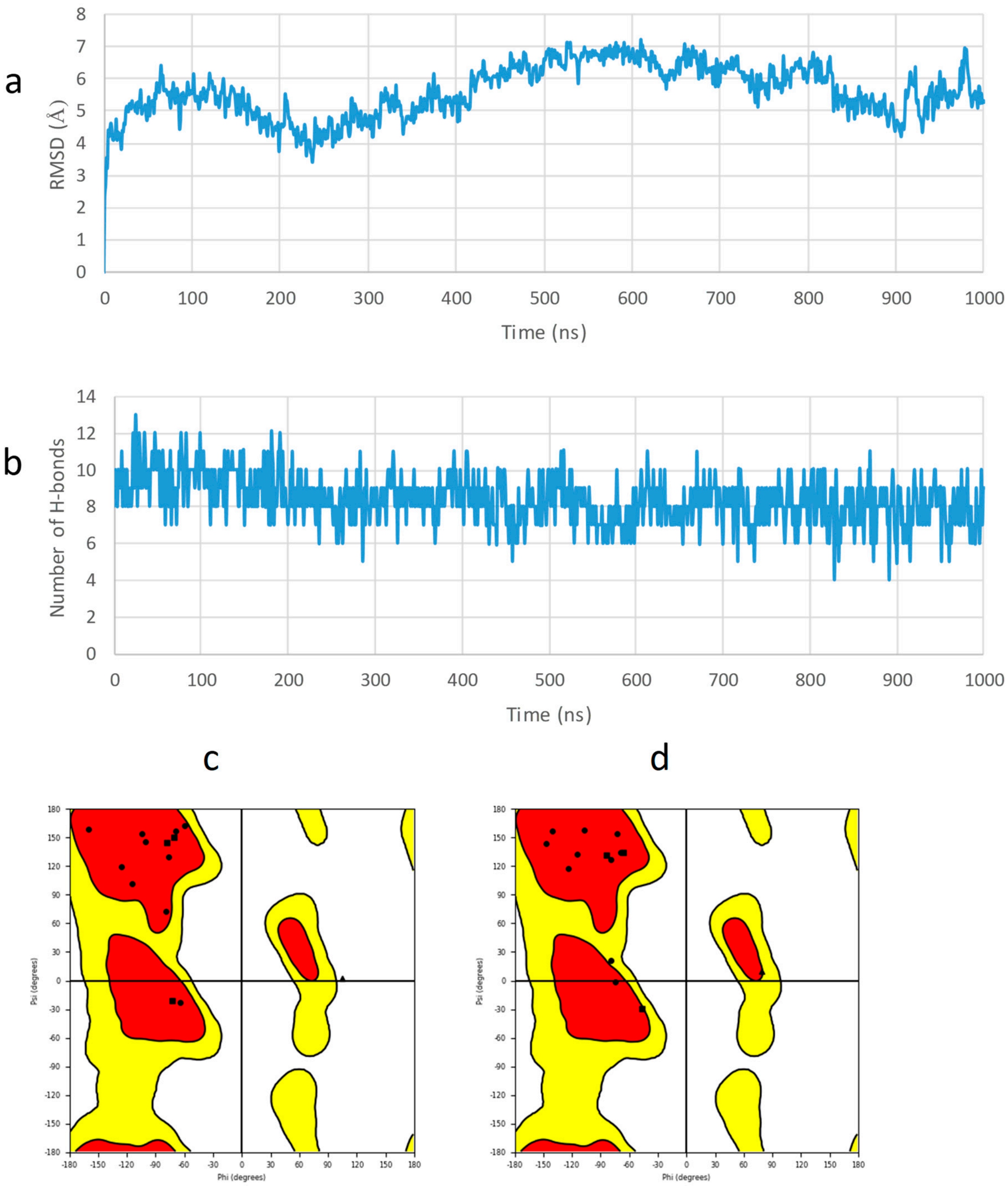

Figure S24. The 1000-ns MD simulation of the SFTI-1–zidovudine conjugate in the POPC membrane model: the RMSD of the α-carbon atoms plotted against the simulation time (a), the number of hydrogen bonds within the peptide plotted against the simulation time (b), the Ramachandran plot of the peptide at 0 ns (c) and at 1000 ns (d) – starting from the top of the membrane.

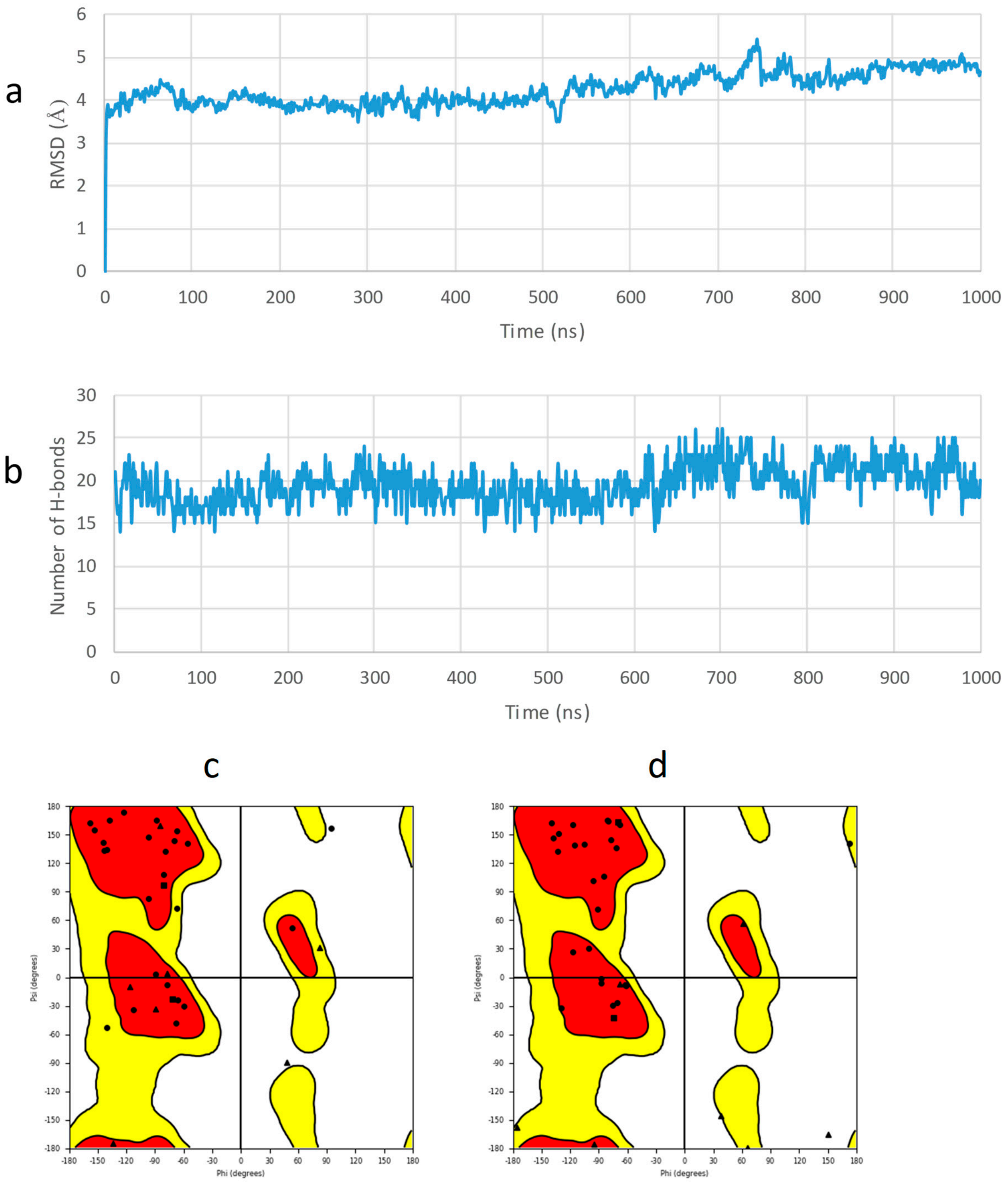

Figure S25. The 1000-ns MD simulation of the MCoTI-II–zidovudine conjugate in the POPC membrane model: the RMSD of the α-carbon atoms plotted against the simulation time (a), the number of hydrogen bonds within the peptide plotted against the simulation time (b), the Ramachandran plot of the peptide at 0 ns (c) and at 1000 ns (d) – starting from the top of the membrane.
